# MTAP loss in gastrointestinal cancers is associated with CDKN2A deletion, poor prognosis in gastric carcinoma, and potential relevance for PRMT5-targeted therapy

**DOI:** 10.1038/s41598-026-51370-9

**Published:** 2026-05-13

**Authors:** Su Ir Lyu, Karl Knipper, Caroline Fretter, Eleni Tzitzili, Adrian Georg Simon, Hakan Alakus, Aylin Pamuk, Christiane J. Bruns, Thomas Schmidt, Felix C. Popp, Alexander Quaas

**Affiliations:** 1 Faculty of Medicine and University Hospital of Cologne, Institute of Pathology, University of Cologne, Cologne, Germany; 2 Faculty of Medicine and University Hospital of Cologne, Department of General, Visceral, Thoracic and Transplantation Surgery, University of Cologne, Cologne, Germany; 3 Institut für Allgemeine Pathologie und Pathologische Anatomie Uniklinik Köln, Kerpener Str. 62, 50937 Cologne, Germany

**Keywords:** MTAP-loss, Esophageal cancer, Gastric cancer, Pancreatic cancer, PRMT5-inhibitor, Cancer, Gastroenterology, Oncology

## Abstract

Gastrointestinal cancers account for a substantial share of global cancer burden. We assessed the significance of MTAP loss across 1545 resected tumors (cholangiocarcinoma, esophageal and gastric adenocarcinoma, pancreatic ductal adenocarcinoma). MTAP protein was evaluated by immunohistochemistry compared with CDKN2A status by fluorescence in situ hybridization and with overall survival. Immunohistochemical MTAP loss consistently co-occurred with homozygous CDKN2A deletion and was present in 25.5% of cholangiocarcinomas, 9.3% of esophageal adenocarcinomas, 10.3% of gastric carcinomas, and 30.2% of pancreatic adenocarcinomas. In gastric carcinomas, MTAP loss was associated with worse survival (*p* = 0.024), particularly in those who underwent primary surgery (*p* = 0.008). MTAP loss proved to be an independent factor for worse overall survival in patients with gastric adenocarcinoma. 10% of esophageal adenocarcinomas showed intratumoral heterogeneity, but we did not see intertumoral heterogeneity between the primary tumor and corresponding lymph node metastases. These data indicate that a considerable subset of gastrointestinal cancers exhibits MTAP loss, highlight prognostic relevance in gastric carcinoma, and delineate a potentially actionable subgroup for PRMT5 inhibitor therapy.

## Introduction

Gastrointestinal cancers account for 26% of global cancer incidence and 35% of all cancer-related deaths worldwide - pancreatic cancer alone is among the most aggressive cancers with high mortality rates. With an expected increase in cancer mortality in the upcoming years, pancreatic cancer will likely surpass colorectal cancer deaths by 2040^1–3^. Epidemiology varies between the distinct gastrointestinal malignancies, but overall, incidence and cancer-related deaths are expected to increase in the upcoming years^3^. Gastric cancer incidence has been declining in the past decades; however, it remains the 5th most frequently diagnosed cancer and the 3rd leading cause of cancer death worldwide^3^. Esophageal cancer represents the 7th most common cause of cancer morbidity and the 6th most common cause of cancer-related death worldwide^3^. Late diagnosis and advanced cancer stages at the time of diagnosis lead to poor prognosis, particularly among pancreatic and gastric cancers^3^. Meanwhile, cholangiocarcinoma is less common than the previously mentioned malignancies, only accounting for about 3% of cancers arising from the gastrointestinal tract, but the incidence is also steadily increasing^4^. A better understanding of tumor biology may enable the identification and targeting of distinct molecular pathways important for tumor survival.

5′-Methylthioadenosine phosphorylase (MTAP) is a key enzyme involved in polyamine metabolism and recycles methylthioadenosine (MTA) to adenine and methionine, and salvages them^5,6^. MTAP loss has been observed in various cancer entities, including gastro-intestinal adenocarcinomas, neuroendocrine neoplasms, Hodgkin lymphoma, mesothelioma, urothelial neoplasms, squamous cell carcinomas, and types of sarcomas^7,8^. MTAP loss leads to dependence of the cancer cell on *de novo* purine synthesis, making it more susceptible to inhibitors of *de novo* purine synthesis such as antifolates^9,10^. MTAP loss, moreover, leads to MTA accumulation, which inhibits several enzymes, including protein arginine methyltransferase 5 (PRMT5), a regulatory protein involved in genome organization, transcription, and cell cycle regulation^11^. The genetic depletion of PRMT5 has previously been linked to decreased cancer cell viability through G1 cell cycle arrest and apoptosis, and PRMT5 has recently been established as a novel epigenetic antineoplastic target^8^. Cells with MTAP loss and subsequent MTA accumulation, leading to PRMT5 inhibition, have been observed to exhibit increased sensitivity through synergistic effects to these novel therapeutic PRMT5 inhibitors, resulting in growing research interest in the interaction of MTAP and PRMT5 inhibitors^8,12^.

The MTAP gene is located at 9p21.3, about 100 kb apart from the tumor suppressor gene *CDKN2A*, which is deleted in up to 15% of cancers itself^7,13^. Co-deletions of MTAP and CDKN2A are common due to their proximity and have been described in various cancer entities^6,14^. About 80–90% of cases with homozygous 9p21.3 deletion exhibit co-deletion of MTAP and CDKN2A^13^.

Here, we aim to elucidate the frequency and clinical significance of MTAP loss in gastrointestinal malignancies to evaluate the potential therapeutic value of PRMT5 inhibitors in these highly lethal cancers.

## Materials and methods

### Patients and tumor samples

All Patients included in this study (*n* = 1545) were diagnosed with either esophageal adenocarcinoma (*n* = 701), gastric carcinoma (*n* = 468), cholangiocarcinoma (*n* = 55), or pancreatic ductal adenocarcinoma (*n* = 321). Written informed consent for participation in the tissue and data bank was obtained from each patient, and samples were collected prospectively and analyzed retrospectively. All patients with esophageal adenocarcinoma, gastric carcinoma, and cholangiocarcinoma were treated at the University Hospital of Cologne. The included patients with pancreatic adenocarcinomas were included in the multicentric PANCALYZE study. Here, the samples were transferred and analyzed at the University Hospital of Cologne. The study received approval from the Ethics Committee (ethics committee numbers: 16–230 and 21-1146). The conduction of the study followed the Declaration of Helsinki. Tumor stages were assessed following the 7th edition of the Union for International Cancer Control. Overall survival was defined as the time from the date of resection until patient death or loss of follow-up.

After appropriate sample tissue quality was ensured by an experienced pathologist, tissue cylinders of 1.2 millimeters were punched out of the samples with a semi-automated precision instrument and transferred to a paraffin-embedded tissue microarray. Slices of 4 μm of the microarray were obtained and further analyzed as explained in the following paragraph.

### Immunohistochemistry

Immunohistochemical staining was conducted using the staining system Leica BOND-MAX with Leica Bond Polymer Refine Detection Kit (Leica Biosystems, Wetzlar, Germany). The rabbit monoclonal antibody against human MTAP was used as indicated by the manufacturer (E5R1I, Cell Signaling Technology, Danvers, USA).

The interpretation of the stained samples was conducted by two experienced pathologists (A.Q. and S.L.). The staining intensity of tumorous cells was compared to the adjacent normal stromal cells of each section. Staining intensity was divided into none (MTAP loss) and weak to strong (retained MTAP). Complete MTAP loss was defined as completely negative staining, and MTAP staining in the adjacent normal tissue was required. Tumor cells with no staining for MTAP but a lack of staining in the adjacent stromal cells were considered inconclusive and excluded.

### Fluorescence in situ hybridization

For fluorescence in situ hybridization the Zytolight SPEC CDKN2A/CEN 9 Dual Color Probe (ZytoVision, Bremerhaven, Germany), which is used to detect deletion of the *CDKN2A* gene and classical satellite DNA III on chromosome 9, was used. FISH analyses were performed as described previously^15^. The fully automated upright fluorescence microscope Leica DM5500 B (Leica Biosystems, Wetzlar, Germany) was used, and imaging was performed with a JVC KY‑F75 digital camera (JVCKENWOOD USA Corporation, Long Beach, USA). The absence of green staining with retained red staining was defined as CDKN2A loss. Normal tissue was used as an internal control.

### Statistical analysis

Statistical analyses were performed with IBM SPSS Statistics (Version 29.0.1.1, Armonk, USA) and R (version 4.3.2, R Foundation for Statistical Computing, Vienna, Austria). P-values below 0.05 were considered statistically significant. Statistical analyses of qualitative variables were performed using the chi-square test. Survival analyses were conducted with Kaplan-Meier curves and log-rank tests. Interdependencies between patient survival and clinicopathological values were analyzed using univariable and multivariable Cox regression analyses. Only variables with P-values below 0.2 in univariable Cox regression analyses were transferred to multivariable Cox regression analyses.

## Results

### Occurrence of MTAP loss in patients with esophageal adenocarcinoma, gastric carcinoma, cholangiocarcinoma, or pancreatic adenocarcinoma

We included a total of 1545 patients in this study. Each of these patients had been diagnosed with either esophageal adenocarcinoma (n = 701), gastric carcinoma (n = 468), cholangiocarcinoma (n = 55), or pancreatic adenocarcinoma (*n* = 321). All clinicopathological values are depicted in Tables 1, 2, 3 and 4. Both primarily operated and neoadjuvant-treated patients were included (neoadjuvant-treated patients: esophageal adenocarcinoma n = 481 (68.6%), gastric carcinoma n = 188 (40.2%), pancreatic adenocarcinoma n = 17 (5.3%)).

**Table 1:** General clinicopathological values of the study population with **cholangiocarcinoma** and patients with MTAP loss versus retained MTAP.

Characteristic	Total	MTAP		*p*-value
		Loss	Retained	
	n (%)	n (%)	n (%)	
No. of patients	55 (100)	14 (25.5)	41 (74.5)	
**Age**				
Under 65 65 and above	14 (25.5) 41 (74.5)	4 (0.29) 10 (71.4)	10 (24.4%) 31 (75.6%)	1
**Sex**				
Male Female	33 (60) 22 (40)	8 (57.1) 6 (42.9)	25 (61) 16 (39)	1
**Adjuvant therapy**				
No Yes	30 (54.4) 25 (45.5)	11 (78.6) 3 (21.4)	19 (46.3) 22 (53.7)	0.075
**Type**				
Intrahepatic Extrahepatic	37 (67.3) 18 (32.7)	7 (50) 7 (50)	30 (73.2) 11 (26.8)	0.206
**Gross features**				
Mass-forming Periductal Intraductal Extrahepatic	24 (43.6) 9 (16.4) 3 (5.5) 19 (34.5)	5 (35.7) 2 (14.3) 0 (0) 7 (50)	19 (46.4) 7 (17) 3 (7.3) 12 (29.3)	0.45
**Histological type**				
Small duct type Large duct type Extrahepatic	29 (52.7) 7 (12.7) 19 (34.5)	7 (50) 0 (0) 7(50)	22 (53.7) 7 (17) 12 (29.3)	0.156
**Grading**				
1 2 3 4	1 (1.8) 33 (60) 20 (36.4) 1 (1.8)	0 (0) 8 (57.1) 6 (42.9) 0 (0)	1 (2.4) 25 (61) 14 (34.1) 1 (2.4)	0.819
**T **				
1 2 3 4	12 (21.8) 26 (47.3) 11 (20) 6 (11)	3 (21.4) 6 (42.9) 4 (28.6) 1 (7.1)	9 (22) 20 (48.8) 7(17) 5 (12.2)	0.798
**N**				
0 1 or greater	31 (56.4) 24 (43.6)	7 (50) 7 (50)	24 (58.5) 17(41.5)	0.807
**L **				
0 1	8 (14.5) 47 (85.5)	1 (7.1) 13 (92.9)	7 (17.1) 34 (82.9)	0.638
**V**				
0 1	31 (56.4) 24 (43.6)	7 (50) 7 (50)	24 (58.5) 17 (41.5)	0.807
**R **				
0 1 2	41 (74.5) 13 (31.7) 1 (1.8)	11 (78.6) 3 (21.4) 0 (0)	30 (63.2) 10 (24.4) 1 (2.4)	0.81
**Pn**				
0 1	19 (34.5) 36 (65.5)	4 (28.6) 10 (71.4)	15 (36.6) 26 (63.3)	0.827
**M**				
0 1	51 (92.7) 4 (7.3)	12 (85.7) 2 (14.3)	39 (95.1) 2 (4.9)	0.566
**Inflammation**				
Low High	39 (70.9) 16 (29.1)	12 (85.7) 2 (14.3)	27 (65.9) 14 (34.1)	0.284
**Precursor lesions**				
No BLIN (biliary intraepithelial neoplasm) Caroli IPNBD (intraductal papillary neoplasm)	38 (69.1) 14 (25.5) 1 (1.8) 2 (3.6)	9 (64.3) 5 (35.7) 0 (0) 0 (0)	29 (70.7) 9 (22) 1 (2.4) 2 (4.9)	0.601
**Liver disease**				
No Pathological Not assessable	27 (49.1) 19 (34.5) 9 (16.4)	7 (50) 5 (35.7) 2 (14.3)	20 (48.8) 14 (34.1) 7 (17.1)	0.97
**Cirrhosis**				
Negative Positive Not assessable	40 (72.7) 6 (10.9) 9 (16.4)	11 (78.6) 1 (7.1) 2 (14.3)	29 (70.7) 5 (12.2) 7 (17.1)	0.826
**Steatohepatitis**				
Negative Positive Not assessable	43 (78.2) 8 (14.5) 4 (7.3)	10 (71.4) 3 (21.4) 1 (7.1)	33 (80.4) 5 (12.2) 3 (7.3)	0.697
**Claudin 18.2**				
Negative Positive (> 75%)	51 (92.7) 4 (7.3)	12 (85.7) 2 (14.3)	39 (95.1) 2 (4.9)	0.566

G: Grading, L: lymph vessel infiltration, M: metastatic status, Pn: perineural infiltration, pN: pathological lymph node status, pT: pathological tumor status, R: complete resection status, V: blood vessel infiltration.

**Table 2:** General clinicopathological values of the study population with **esophageal adenocarcinoma** and patients with MTAP loss versus retained MTAP.

Characteristic	Total	MTAP		*p*-value
		Loss	Retained	
	n (%)	n (%)	n (%)	
No. of patients	701 (100)	65 (9.3)	636 (90.7)	
**Age**				
Under 65 65 and above	384 (54.8) 317 (45.2)	41 (63.1) 24 (36.9)	343 (53.9) 293 (46.1)	0.2
**Sex**				
Male Female	614 (87.6) 87 (12.4)	61 (93.8) 4 (6.2)	553 (86.9) 83 (13.1)	0.159
**Neoadjuvant therapy**				
No Yes	220 (31.4) 481 (68.6)	22 (33.8) 43 (66.2)	198 (31.1) 438 (68.9)	0.757
**pT **				
1 2 3 4	125 (17.8) 111 (15.8) 442 (63.1) 23 (3.3)	15 (23.1) 12 (18.5) 37 (56.9) 1 (1.5)	110 (17.3) 99 (15.6) 405 (63.7) 22 (3.5)	0.474
**pN**				
0 1 or greater	274 (39.1) 427 (60.9)	28 (43.1) 37 (56.9)	246 (38.7) 390 (61.3)	0.576
**L **				
0 1 2	321 (45.8) 268 (38.2) 112 (16)	31 (47.7) 22 (33.8) 12 (18.5)	290 (45.6) 246 (38.7) 100 (15.7)	0.707
**V**				
0 1 2	522 (74.4) 72 (10.3) 107 (15.3)	45 (69.2) 9 (13.8) 11 (16.9)	477 (75) 63 (9.9) 96 (15.1)	0.526
**Pn**				
0 1 2	452 (64.5) 145 (20.7) 104 (14.8)	38 (58.5) 16 (24.6) 11 (16.9)	414 (65.1) 129 (20.3) 93 (14.6)	0.563
**G**				
1 2 3 4	1 (0.1) 127 (18.1) 135 (19.3) 2 (0.3)	0 (0) 15 (23.1) 12 (18.5) 1 (1.5)	1 (0.2) 112 (17.6) 123 (19.3) 1 (0.2)	0.259
**Claudin 18.2**				
Negative Positive (> 75%)	481 (68.6) 166 (23.7)	46 (70.8) 16 (24.6)	435 (68.4) 150 (23.6)	1
**Y chromosome p-arm**				
0 1	330 (47.1) 228 (32.5)	38 (58.5) 20 (30.8)	292 (45.9) 208 (32.7)	0.367
**Her2 IHC**				
0 1 2 3	564 (80.5) 1 (0.1) 26 (3.7) 44 (6.3)	49 (75.4) 0 (0) 4 (6.2) 6 (9.2)	515 (81) 1 (0.2) 22 (3.5) 38 (6)	0.479

G: Grading, IHC: immunohistochemistry, L: lymph vessel infiltration, Pn: perineural infiltration, pN: pathological lymph node status, pT: pathological tumor status, V: blood vessel infiltration.

**Table 3:** General clinicopathological values of the study population with **gastric carcinoma** and patients with MTAP loss versus retained MTAP.

Characteristic	Total	MTAP		*p*-value
		Loss	Retained	
	n (%)	n (%)	n (%)	
No. of patients	468 (100)	48 (10.3)	420 (89.7)	
**Age**				
Under 65 65 and above	224 (47.9) 244 (52.1)	17 (35.4) 31 (64.6)	207 (49.3) 213 (50.7)	0.068
**Sex**				
Male Female	312 (66.7) 156 (33.3)	32 (66.7) 16 (33.3)	280 (66.7) 140 (33.3)	1
**Neoadjuvant therapy**				
No Yes	280 (59.8) 188 (40.2)	31 (64.6) 17 (35.4)	249 (59.3) 171 (40.7	0.478
**pT**				
1 2 3 4	68 (14.5) 56 (12.0) 203 (43.4) 141 (30.1)	6 (12.5) 3 (6.3) 26 (54.2) 13 (27.1)	62 (14.8) 53 (12.6) 177 (42.1) 128 (30.5)	0.259
**pN**				
0 1 or greater	172 (36.8) 296 (63.2)	17 (35.4) 31 (64.6)	155 (36.9) 265 (63.1)	0.839
**L**				
0 1 Unknown	128 (27.4) 227 (48.5) 113 (24.1)	10 (20.8) 25 (52.1) 13 (27.1)	118 (28.1) 202 (48.1) 100 (23.8)	0.331
**V**				
0 1 Unknown	208 (44.4) 64 (13.7) 196 (41.9)	18 (37.5) 7 (14.6) 23 (47.9)	190 (45.2) 57 (13.6) 173 (41.2)	0.580
**Pn**				
0 1 Unknown	24 (5.1) 20 (4.3) 424 (90.6)	1 (2.1) 2 (4.2) 45 (93.7)	23 (5.5) 18 (4.2) 379 (90.2)	0.445
**G**				
1 2 3 4 Unknown/not applicable	2 (0.4) 118 (25.2) 199 (42.4) 3 (0.6) 146 (31.2)	0 (0) 17 (35.4) 18 (37.5) 0 (0) 13 (27.1)	2 (0.5) 101 (24.0) 181 (43.1) 3 (0.7) 133 (31.7)	0.421

G: Grading, L: lymph vessel infiltration, Pn: perineural infiltration, pN: pathological lymph node status, pT: pathological tumor status, V: blood vessel infiltration.

**Table 4:** General clinicopathological values of the study population with **pancreatic adenocarcinoma** and patients with MTAP loss versus retained MTAP.

Characteristic	Total	MTAP		*p*-value
		Loss	Retained	
	n (%)	n (%)	n (%)	
No. of patients	321 (100)	97 (30.2)	224 (69.8)	
**Age**				
Under 65 65 and above	103 (32.1) 218 (67.9)	37 (38.1) 60 (61.9)	66 (29.5) 158 (70.5)	0.126
**Sex**				
Male Female	157 (48.9) 164 (51.1)	50 (51.5) 47 (48.5)	107 (47.8) 117 (52.2)	0.534
**Neoadjuvant therapy**				
No Yes	304 (94.7) 17 (5.3)	92 (94.8) 5 (5.2)	212 (94.6) 12 (5.4)	0.941
**pT **				
1 2 3 4	22 (6.9) 121 (37.7) 171 (53.3) 7 (2.2)	5 (5.2) 35 (36.1) 56 (57.7) 1 (1)	17 (7.6) 86 (38.4) 115 (51.3) 6 (2.7)	0.563
**pN**				
0 1	92 (28.7) 229 (71.3)	28 (28.9 69 (71.1)	64 (28.6) 160 (71.4)	0.957
**L **				
0 1 Unknown	122 (38) 196 (61.1) 3 (0.9)	34 (35.1) 62 (63.9) 1 (1.0)	88 (39.3) 134 (59.8) 2 (0.9)	0.477
**V**				
0 1 Unknown	220 (69.6) 95 (30.1) 6 (0.3)	66 (68) 30 (30.9) 1 (1.0)	154 (68.8) 65 (29) 5 (2.2)	0.773
**Pn**				
0 1 Unknown	77 (24) 232 (72.3) 12 (3.7)	19 (19.6) 77 (79.4) 1 (1.0)	58 (25.9) 155 (69.2) 11 (4.9)	0.162
**G**				
0 1 2 3 4 Unknown	7 (2.2) 10 (3.1) 156 (48.6) 138 (43) 2 (0.6) 8 (2.5)	1 (1.0) 1 (1.0) 53 (54.6) 41 (42.3) 1 (1.0) 0 (0)	6 (2.7) 9 (4.0) 103 (46.0) 97 (43.3) 1 (0.5) 8 (3.6)	0.397

G: Grading, L: lymph vessel infiltration, Pn: perineural infiltration, pN: pathological lymph node status, pT: pathological tumor status, V: blood vessel infiltration.

**Fig. 1:**
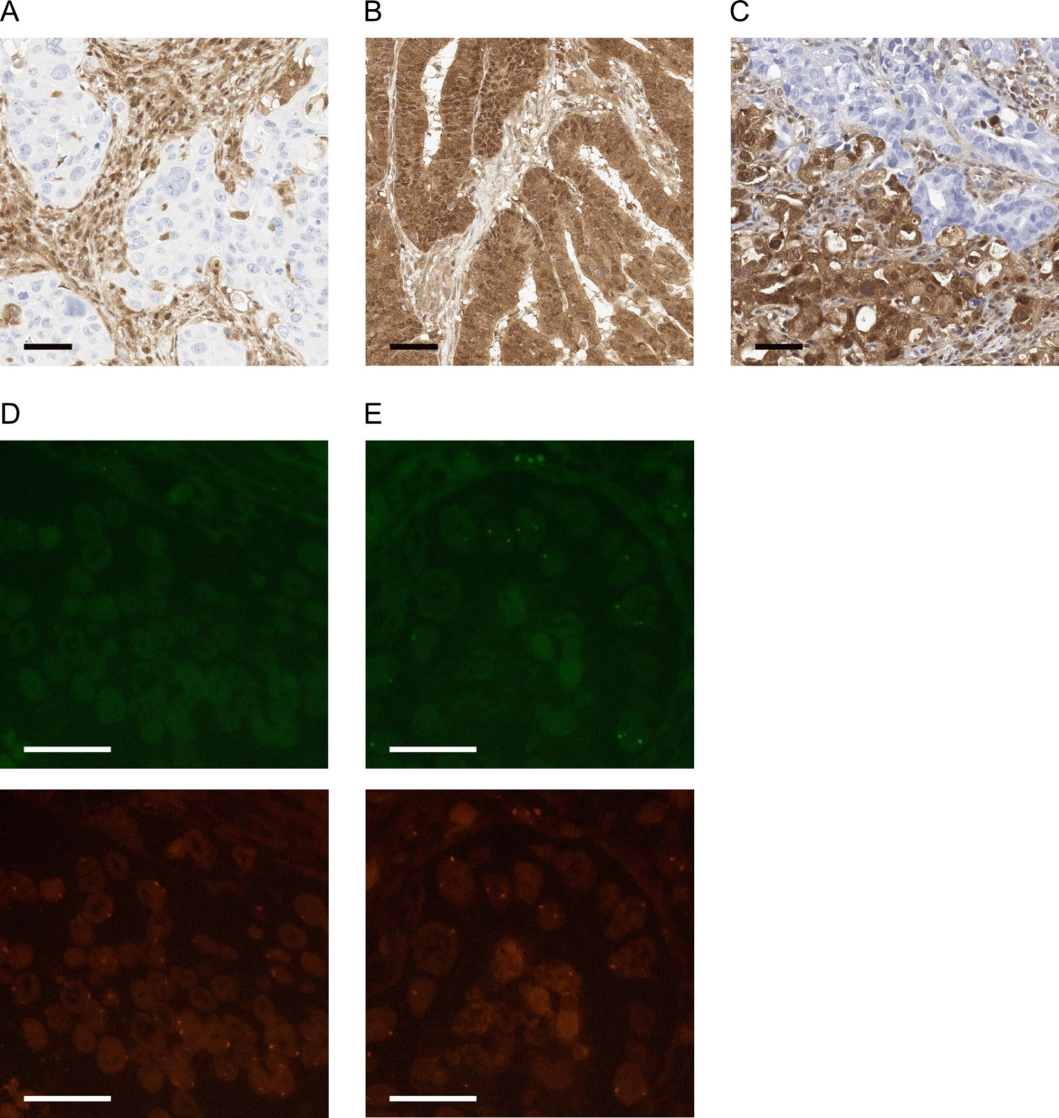
Exemplary pictures of immunohistochemical MTAP stainings in esophageal adenocarcinoma with (**A**) MTAP loss, (**B**) retained MTAP expression, and (**C**) heterogeneous MTAP expression. Furthermore, representative pictures of *CDKN2A* FISH stainings in esophageal adenocarcinoma with (**D**) *CDKN2A* loss and (**E**) retained *CDKN2A* signal (green: p16, red: centromere 9). Black sidebar: 50 μm, white sidebar: 10 μm.

Tumor samples were immunohistochemically stained for MTAP (Fig. 1A,B). MTAP loss was present in 25.5% of cholangiocarcinoma, 9.3% of esophageal adenocarcinomas, 10.3% of gastric carcinomas, and 30.2% of pancreatic adenocarcinomas. We then divided our cohort into samples with MTAP loss and retained MTAP and compared general clinicopathological values among these subgroups for each tumor entity. (Tables 1, 2, 3 and 4) Here, we did not identify any significant correlation between clinicopathological values and MTAP loss. Moreover, MTAP loss did not correlate with higher or lower tumor stages.

### MTAP loss correlates with worse patient survival in patients with gastric carcinoma

**Fig. 2:**
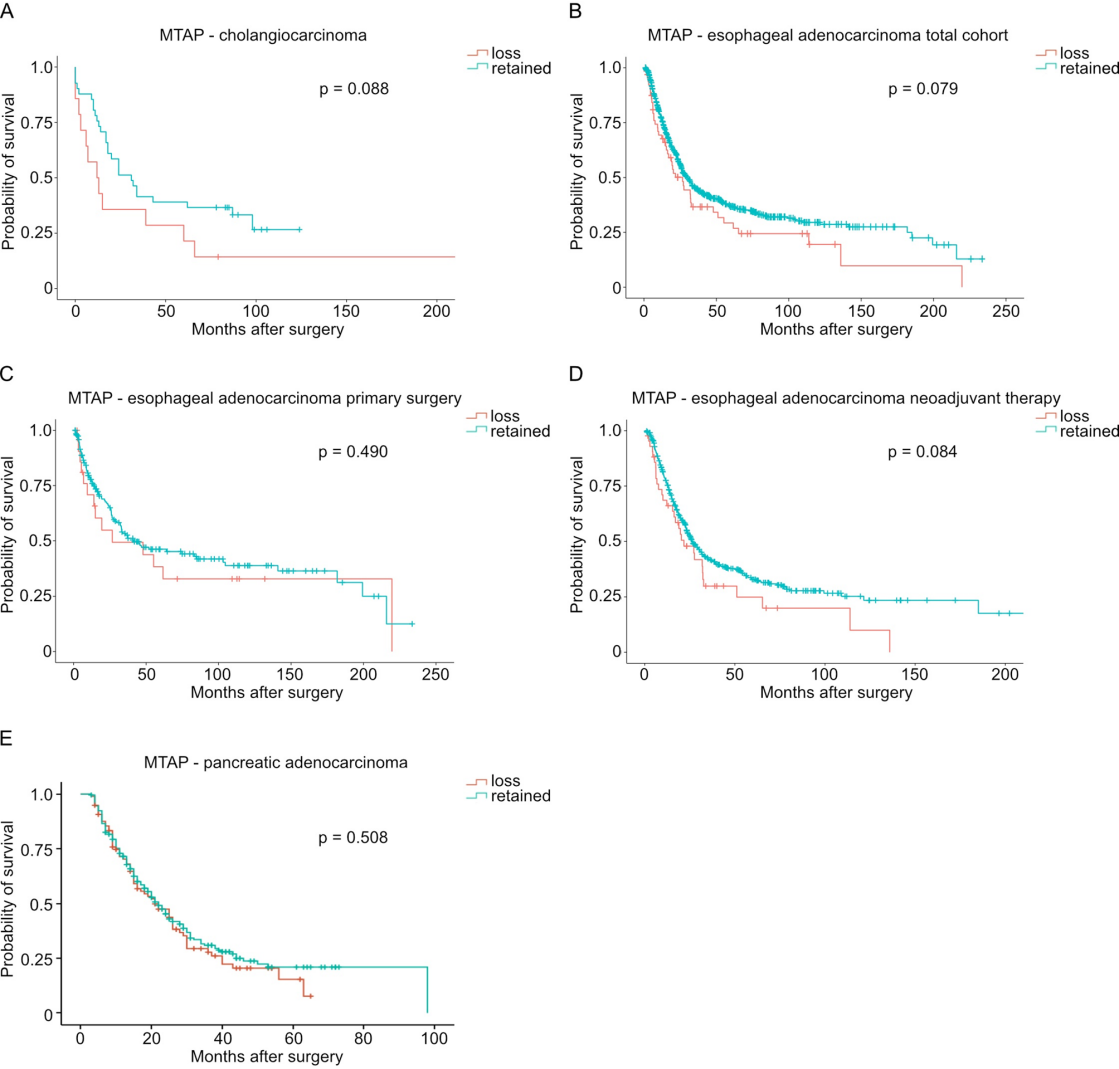
Kaplan–Meier curves of the overall survival depending on MTAP loss-status for (**A**) patients with cholangiocarcinoma (n(MTAP loss) = 14, n(MTAP retained) = 41, *p* = 0.088), (**B**) the total cohort of patients with esophageal adenocarcinoma (n(MTAP loss) = 65, n(MTAP retained) = 636, *p* = 0.079), (**C**) patients with esophageal adenocarcinoma treated with primary surgery (n(MTAP loss) = 22, n(MTAP retained) = 198, *p* = 0.490), (**D**) patients with esophageal adenocarcinoma treated neoadjuvantly (n(MTAP loss) = 43, n(MTAP retained) = 438, *p* = 0.084), and (**E**) patients with pancreatic adenocarcinoma (n(MTAP loss) = 97, n(MTAP retained) = 224, *p* = 0.508).

**Fig. 3:**
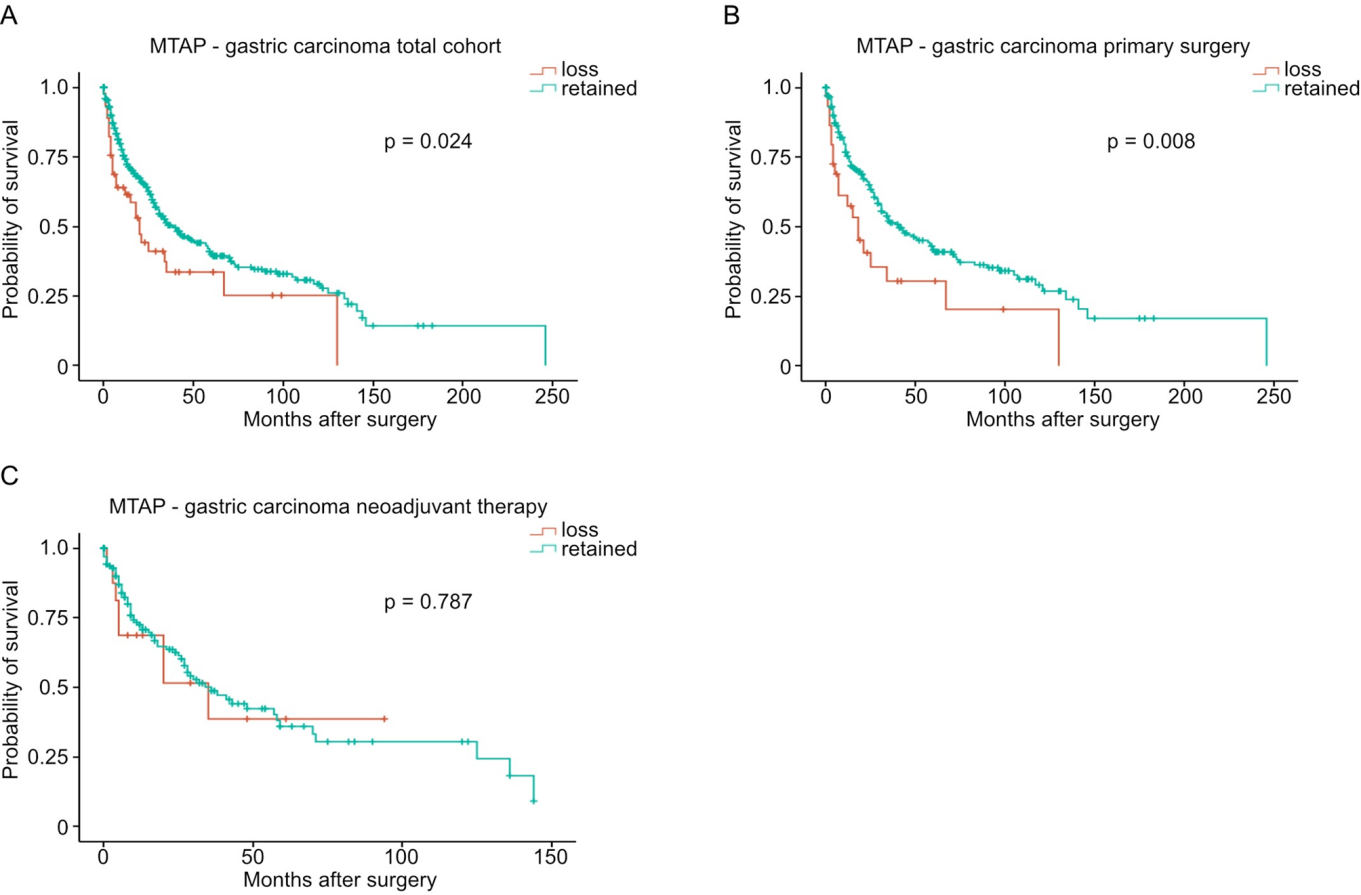
Kaplan–Meier curves of the overall survival depending on MTAP loss-status for (**A**) the total cohort of patients with gastric carcinoma (n(MTAP loss) = 48, n(MTAP retained) = 420, *p* = 0.024), (**B**) patients treated with primary surgery (n(MTAP loss) = 31, n(MTAP retained) = 249, *p* = 0.008), and (**C**) patients treated neoadjuvantly (n(MTAP loss) = 17, n(MTAP retained) = 171, *p* = 0.787).

We then performed survival analyses as outlined in Figs. 2 and 3. There was no statistically significant difference in survival in patients with or without MTAP in cholangiocarcinoma, esophageal adenocarcinoma, or pancreatic adenocarcinoma (Fig. 2). Among patients with gastric carcinoma, loss of MTAP expression was associated with significantly worse survival (*p* = 0.024, Fig. 3A), particularly in those who underwent primary surgery (*p* = 0.008, Fig. 3B), whereas no significant association was observed in patients who received neoadjuvant therapy (*p* = 0.787, Fig. 3C).

### MTAP loss is an independent factor for worse patient survival in patients with gastric adenocarcinoma

Since the MTAP loss-status showed a significant impact on patient survival in our gastric carcinoma cohort, we conducted univariable Cox regression analyses in this patient cohort. Here, a retained MTAP signal was a factor for better patient survival (HR = 0.637, 95% CI = 0.428–0.948, *p* = 0.026). Furthermore, pT-, pN-, L-, V-status and Grading proved to be predictors for a worse patient overall survival (pT: HR = 1.668, 95% CI = 1.436–1.938, *p* ≤ 0.001; pN: HR = 1.573, 95% CI = 1.410–1.754, *p* ≤ 0.001; L: HR = 2.856, 95% CI = 1.937–4.212, *p* ≤ 0.001; V: HR = 2.359, 95% CI = 1.561–3.564, *p* ≤ 0.001; G: HR = 1.425, 95% CI = 1.050–1.934, *p* = 0.023).

To further investigate possible interdependencies, we conducted multivariable Cox regression analysis. The analysis could confirm a retained MTAP signal as an independent factor for better patient survival in our total cohort of patients with gastric adenocarcinoma (HR = 0.404, 95% CI = 0.200-0.816, *p* = 0.011, Table 5). Furthermore, a higher lymph node status was a significant, independent factor for worse overall survival in patients with gastric carcinoma (HR = 1.672, 95% CI = 1.281–2.182, *p* ≤ 0.001, Table 5).

**Table 5:** Multivariable Cox regression analysis for the total cohort of patients with gastric adenocarcinoma.

Characteristic	Borders	Hazard ratio	95% Confidence interval	*p*-value
pT	≥ 2 versus 1	1.115	0.757–1.642	0.582
pN	≥ 1 versus 0	1.672	1.281–2.182	**< 0.001**
L	1 versus 0	1.874	0.955–3.677	0.068
V	1 versus 0	1.086	0.602–1.962	0.783
G	≥ 2 versus 1	1.025	0.580–1.812	0.931
MTAP	Retained versus loss	0.404	0.200–0.816	**0.011**

Bold print marks p-values below 0.05. G: Grading, L: lymph vessel infiltration, pN: pathological lymph node status, pT: pathological tumor status, V: blood vessel infiltration.

### MTAP expression is mainly homogeneous in esophageal adenocarcinoma

To assess the heterogeneity of the immunohistochemical MTAP loss status, we performed immunohistochemical MTAP stainings on 20 whole tumor sections of our patient cohort with esophageal adenocarcinoma. Following this, the stainings were assessed by two independent pathologists regarding the heterogeneity of MTAP loss. Here, two tumors presented a heterogeneous MTAP expression (10% intratumoral heterogeneity, Fig. 1C). 90% of the tumors showed a homogeneous MTAP expression level throughout the whole tumor sections. Furthermore, we compared the MTAP expression level between the primary tumor and the corresponding lymph node metastases in our cohort with esophageal adenocarcinoma (n(paired samples) = 210; 190 samples with retained MTAP and 20 with MTAP loss). The same MTAP loss status could be detected in all paired tumors. In detail, paired samples of 190 patients showed a retained MTAP expression in the primary tumor and lymph node metastasis, as well as 20 patients showed MTAP loss in both, paired primary tumor and lymph node metastasis (no intertumoral heterogeneity).

### MTAP loss co-occurs with CDKN2A deletion

MTAP loss occurs due to co-deletion during CDKN2A loss^14^. Therefore, we aimed to verify whether the lack of the protein MTAP, detectable by immunohistochemistry, co-occurs with a deletion of CDKN2A at the genomic level using fluorescence in situ hybridization (FISH). Two microarrays of our esophageal adenocarcinoma cohort containing 148 patients were additionally examined with fluorescence in situ hybridization for CDKN2A and compared with immunohistochemical stainings for MTAP (Fig. 1D + E). MTAP loss was detected in a total of eleven tumor samples, out of which ten showed a CDKN2A loss, and one sample was not interpretable. MTAP loss and CDKN2A loss were therefore found to overlap.

### MTAP loss doesn’t co-occur with other prognostically and therapeutically important markers

Co-occurrences of several therapeutically or prognostically relevant markers could lead to combined therapy options or patient subgroups with a need for more frequent follow-up exams. Therefore, we related the occurrence of MTAP loss with previously assessed and published markers, like HER2, Y chromosome, or Claudin 18.2, in our group of the same patient cohort with esophageal adenocarcinoma and cholangiocarcinoma^16–18^. We could not see an association of Claudin 18.2-positivity with the occurrence of MTAP loss, neither in cholangiocarcinoma (*p* = 0.566), nor in esophageal adenocarcinoma (*p* = 1.000, Tables 1 and 2). Furthermore, there was no correlation between MTAP loss and Y chromosome loss (*p* = 0.367) or HER2 positivity in esophageal adenocarcinoma (*p* = 0.367, Table 2).

## Discussion

In this study, we analyzed the epidemiology and clinical implications of MTAP/CDKN2A gene loss in gastrointestinal cancers. Loss of MTAP was observed in 25.5% of cholangiocarcinoma, 9.3% of esophageal carcinomas, 10.3% of gastric carcinomas, and 30.2% of pancreatic adenocarcinomas. These findings are largely consistent with previous studies on the prevalence of MTAP expression in gastrointestinal tumors, thereby supporting the validity of our dataset and analysis^7,19^. MTAP loss furthermore co-occurred with CDKN2A loss. The two genes are located about 100 kb apart, making MTAP a valid surrogate marker for CDKN2A which is deleted in about 15% of cancers and has been found to be associated with poorer survival itself^20^. Our analysis did not reveal predictors for MTAP loss or clinical implications of MTAP loss on cancer grade. However, in patients with gastric carcinoma, loss of MTAP was found to be associated with significantly worse survival (*p* = 0.024), particularly among those who underwent primary surgery (*p* = 0.008). Moreover, retained MTAP expression remained an independent predictor of better overall survival in gastric carcinoma in multivariable analysis (HR = 0.404, 95% CI = 0.200–0.816, *p* = 0.011). These observations are in line with reports from other tumor entities, in which MTAP loss has also been associated with worse patient survival, including Ewing sarcoma, urothelial bladder carcinoma, and non-small cell lung cancer^21–23^. Nevertheless, no impact of the MTAP loss status on patient survival could be observed in pancreatic, esophageal adenocarcinoma, and cholangiocarcinoma in our study. Accordingly, previous studies could not find a significant impact of the MTAP loss status on patient survival in cohorts with pancreatic cancer and cholangiocarcinoma^24^.

The prognostic effect of MTAP loss in gastric carcinoma deserves particular attention. Our findings are in line with a previous study in gastric carcinoma that also reported an association between reduced MTAP expression and worse patient survival, supporting the biological and clinical relevance of MTAP loss in this entity^25^. By contrast, such a prognostic association has not been consistently observed across all gastrointestinal tumor types, including recent GI-wide analyses, suggesting that the biological consequences of MTAP loss may be context dependent^19^. One possible explanation is that in gastric carcinoma, MTAP loss and the associated 9p21 alterations may be linked more closely to tumor aggressiveness, invasive behavior, or specific metabolic vulnerabilities than in other gastrointestinal cancers. Although the mechanistic basis of this entity-specific association was not addressed in the present study, prior functional data in gastric cancer suggest that MTAP loss may contribute to tumor growth and invasion^25^. From a clinical perspective, our findings indicate that MTAP status may have value for risk stratification in gastric carcinoma and may help define a subgroup of patients that could be of interest for future biomarker-driven therapeutic strategies, including approaches targeting PRMT5-related dependencies. However, these implications require validation in independent cohorts and prospective studies.

MTAP gene loss is becoming of increasing interest in the research community due to new targeted cancer therapies directed against PRMT5. Various PRMT5 inhibitors have been established in xenograft models and have shown significant tumor lethality in MTAP-deleted cells through synergistic inhibition of PRMT5 with MTAP loss-mediated MTA accumulation^26–28^. PRMT5/MTA complex inhibitors, including TNG908, MRTX1719, and AMG193 have been developed and aim to selectively inhibit PRMT5 in MTAP-deleted cells, decreasing toxicity to normal surrounding cells^12^. PRMT5 is an enzyme required for cell cycle progression, and hematopoietic toxicities have previously been observed with nonselective PRMT5 inhibitors^26^. MRTX1719 is one of the currently studied PRMT5 inhibitors, presently in a phase I/II clinical trial in patients with solid tumors with MTAP deletions^26^. Early results suggest complete inhibition of PRMT5 in MTAP-deleted cells without observed hematopoietic stem cell toxicities^26^. These results are promising, and further studies will be required to prove the safety and efficacy of PRMT5 inhibitors in MTAP-deleted solid tumors. Additional PRMT5 inhibitors are currently under investigation in phase I trials, including JNJ-64,619,178, AMG 193, and PF-06939999^29–31^. Considering the substantial proportion of gastrointestinal tumors with MTAP loss in our cohort, further investigation of PRMT5 inhibitors in the gastrointestinal tumor entities examined here appears warranted.

MTAP loss might not be the only predictor for sensitivity to PRMT5 inhibitors^13^. While MTA accumulation is postulated as the main reason for sensitivity to PRMT5 inhibitors in cells with MTAP loss, a study evaluating MTAP loss in glioblastoma suggested lower expression of MTA in vivo due to expression of MTAP in tumor surrounding stroma, highlighting the importance of considering tumor microenvironment^32^. Since this phenomenon has thus far only been observed in glioblastoma, dedicated research on PRMT5 inhibitor efficacy in gastrointestinal tumors could be considered, while keeping in mind that tumor specific microenvironmental changes might influence response to these targeted therapies.

Data specifically addressing intratumoral MTAP heterogeneity in esophageal adenocarcinoma are scarce. In our cohort, heterogeneous expression was observed in only 2 of 20 whole tumor sections, while complete concordance was seen between paired primary tumors and lymph node metastases, suggesting that MTAP is overall a relatively stable biomarker in this entity, although focal heterogeneity may pose a sampling challenge in small biopsy specimens. This aspect is particularly relevant when interpreting biopsy-based or tissue microarray-based analyses, as limited tissue sampling may not fully reflect the MTAP status of the entire tumor.

Overall, a major strength of our study is our large, established cohort, which displays the study’s overall statistical power and permits the analysis of various subgroups. However, some limitations apply to this study. First, the retrospective study design could restrict the ability to generalize the study findings to all patients with the investigated tumor entities. This restriction also applies to the mostly unicentric study design. It should be highlighted that the samples of pancreatic adenocarcinomas were collected in the multicentric PANCALYZE study. Second, we examined mainly the protein expression of MTAP using immunohistochemistry. In some clinical studies, the genomic deletion of MTAP, examined by, for example next-generation sequencing, was used as an inclusion criterion^29^. However, the immunohistochemical MTAP status is an established surrogate marker for genomic MTAP deficiency^33^. In addition, FISH was selected for CDKN2A assessment because the aim of this study was to detect homozygous deletion of the 9p21 locus in a morphologically controlled manner. Given the close genomic proximity of CDKN2A and MTAP, this approach was well suited to examine whether immunohistochemical MTAP loss corresponds to deletion of the neighboring locus^6,14^. By contrast, sequence-based methods may identify point mutations or other alterations that were not essential to the primary objective of this study, which was to assess the concordance between MTAP protein loss and homozygous 9p21 deletion.

In addition, broad integrative molecular profiling was not available across all included tumor entities. Additional biomarkers such as HER2, Claudin18.2, PD-L1/PD-1, FGFR alterations, or KRAS status may help to define biologically and clinically relevant subgroups among MTAP-deficient gastrointestinal cancers. In the present study, correlations with selected previously assessed markers were available only for subcohorts, and no significant associations with HER2, Claudin18.2, or Y chromosome loss were observed. No further mechanistic or therapeutic experiments were conducted. Future studies should therefore integrate MTAP status with broader molecular profiling and functional analyses to further clarify its biological and therapeutic relevance in these tumor entities.

Future clinical studies should address the therapeutic relevance of MTAP loss in these entities. Third, we conducted our immunohistochemical stainings on tissue microarrays. As these microarrays only represent a small part of the whole tumor, potential heterogeneity may have led to sampling bias. However, we conducted immunohistochemical stainings on 20 whole tumor sections of esophageal adenocarcinomas to address this issue. Here, heterogeneous MTAP expression could only be observed in 2 of 20 tumors.

Concluding, we could show that a substantial subset of patients with cholangiocarcinoma, esophageal adenocarcinoma, gastric adenocarcinoma, or pancreatic adenocarcinoma exhibits MTAP loss. Furthermore, the MTAP loss status could be described as an independent risk factor for worse patient survival in gastric carcinoma. The loss of MTAP was highly homogeneous in the primary tumor and corresponding lymph node metastases. Our results suggest that numerous patients with one of these entities are potentially eligible for treatment with PRMT5 inhibitors. Future clinical studies should be conducted to further assess the potential survival benefit of the treatment with PRMT5 inhibitors in these tumor entities.

## Data Availability

The datasets generated and analyzed during the current study are available from the corresponding author upon reasonable request.

## References

[1] Hu, J. X. et al. Pancreatic cancer: A review of epidemiology, trend, and risk factors. *World J. Gastroenterol.* **27**(27), 4298–4321 (2021). 34366606 10.3748/wjg.v27.i27.4298 PMC8316912

[2] Rahib, L., Wehner, M. R., Matrisian, L. M. & Nead, K. T. Estimated projection of US Cancer Incidence and death to 2040. *JAMA Netw. Open.* **4**(4), e214708 (2021). 33825840 10.1001/jamanetworkopen.2021.4708 PMC8027914

[3] Arnold, M. et al. Global burden of 5 major types of gastrointestinal cancer. *Gastroenterology* **159**(1), 335–49e15 (2020). 32247694 10.1053/j.gastro.2020.02.068 PMC8630546

[4] Qurashi, M., Vithayathil, M. & Khan, S. A. Epidemiology of cholangiocarcinoma. *Eur. J. Surg. Oncol.* **51**(2), 107064 (2025). 37709624 10.1016/j.ejso.2023.107064

[5] Liu, T. A., Stewart, T. M. & Casero, R. A. Jr. The synergistic benefit of combination strategies targeting tumor cell polyamine homeostasis. *Int. J. Mol. Sci.* **25**, 15 (2024). 10.3390/ijms25158173 PMC11311409 39125742

[6] Patro, C. P. K. et al. MTAP loss: A possible therapeutic approach for glioblastoma. *J. Transl Med.* **20**(1), 620 (2022). 36572880 10.1186/s12967-022-03823-8 PMC9791736

[7] Gorbokon, N. et al. Prevalence of S-methyl-5’-thioadenosine phosphorylase (MTAP) deficiency in human cancer: A tissue microarray study on 13,067 tumors from 149 different tumor types. *Am. J. Surg. Pathol.* **48**(10), 1245–1258 (2024). 39132873 10.1097/PAS.0000000000002297 PMC11404761

[8] Kryukov, G. V. et al. MTAP deletion confers enhanced dependency on the PRMT5 arginine methyltransferase in cancer cells. *Science* **351**(6278), 1214–1218 (2016). 26912360 10.1126/science.aad5214 PMC4997612

[9] Casero, R. A. Jr., Murray Stewart, T. & Pegg, A. E. Polyamine metabolism and cancer: Treatments, challenges and opportunities. *Nat. Rev. Cancer*. **18**(11), 681–695 (2018). 30181570 10.1038/s41568-018-0050-3 PMC6487480

[10] Tangudu, N. K. et al. De Novo purine metabolism is a metabolic vulnerability of cancers with low p16 expression. *Cancer Res. Commun.* **4**(5), 1174–1188 (2024). 38626341 10.1158/2767-9764.CRC-23-0450 PMC11064835

[11] Stopa, N., Krebs, J. E. & Shechter, D. The PRMT5 arginine methyltransferase: Many roles in development, cancer and beyond. *Cell. Mol. Life Sci.* **72**(11), 2041–2059 (2015). 25662273 10.1007/s00018-015-1847-9 PMC4430368

[12] Hu, M. & Chen, X. A review of the known MTA-cooperative PRMT5 inhibitors. *RSC Adv.* **14**(53), 39653–39691 (2024). 39691229 10.1039/d4ra05497k PMC11650783

[13] Bray, C., Balcells, C., McNeish, I. A. & Keun, H. C. The potential and challenges of targeting MTAP-negative cancers beyond synthetic lethality. *Front. Oncol.* **13**, 1264785 (2023). 37795443 10.3389/fonc.2023.1264785 PMC10546069

[14] Vrugt, B. et al. Deletions of CDKN2A and MTAP detected by copy-number variation array are associated with loss of p16 and MTAP protein in pleural mesothelioma. *Cancers (Basel)* **15**(20), 4978 (2023). 10.3390/cancers15204978 PMC10605896 37894345

[15] Schildhaus, H. U. et al. Definition of a fluorescence in-situ hybridization score identifies high- and low-level FGFR1 amplification types in squamous cell lung cancer. *Mod. Pathol.* **25**(11), 1473–1480 (2012). 22684217 10.1038/modpathol.2012.102 PMC4089812

[16] Moentenich, V. et al. Claudin 18.2 expression in esophageal adenocarcinoma and its potential impact on future treatment strategies. *Oncol. Lett.* **19**(6), 3665–3670 (2020). 32391091 10.3892/ol.2020.11520 PMC7204493

[17] Loeser, H. et al. Y chromosome loss is a frequent event in Barrett’s adenocarcinoma and associated with poor outcome. *Cancers (Basel)* **12**(7), 1743 (2020). 10.3390/cancers12071743 PMC7408596 32629877

[18] Plum, P. S. et al. HER2/neu (ERBB2) expression and gene amplification correlates with better survival in esophageal adenocarcinoma. *BMC Cancer*. **19**(1), 38 (2019). 30621632 10.1186/s12885-018-5242-4 PMC6325716

[19] Mauri, G. et al. Clinicopathological characterisation of MTAP alterations in gastrointestinal cancers. *J. Clin. Pathol.* **78**(3), 195–201 (2025). 38350716 10.1136/jcp-2023-209341 PMC11874331

[20] Doyle, A. et al. The impact of CDKN2A mutations on overall survival in pancreatic adenocarcinoma. *J. Clin. Oncol.* **37**(4_suppl), 278 (2019).30550363

[21] Abrahao-Machado, L. F. et al. Loss of MTAP expression is a negative prognostic marker in Ewing sarcoma family of tumors. *Biomark. Med.* **12**(1), 35–44 (2018). 29243509 10.2217/bmm-2017-0152

[22] Gorbokon, N. et al. Loss of MTAP expression is strongly linked to homozygous 9p21 deletion, unfavorable tumor phenotype, and noninflamed microenvironment in urothelial bladder cancer. *J. Pathol. Clin. Res.* **11**(1), e70012 (2025). 39668577 10.1002/2056-4538.70012 PMC11638363

[23] Su, C. Y. et al. MTAP is an independent prognosis marker and the concordant loss of MTAP and p16 expression predicts short survival in non-small cell lung cancer patients. *Eur. J. Surg. Oncol.* **40**(9), 1143–1150 (2014). 24969958 10.1016/j.ejso.2014.04.017

[24] Ngoi, N. Y. L. et al. Methylthioadenosine phosphorylase genomic loss in advanced gastrointestinal cancers. *Oncologist* **29**(6), 493–503 (2024). 38330461 10.1093/oncolo/oyae011 PMC11144995

[25] Kim, J. et al. Downregulation of methylthioadenosin phosphorylase by homozygous deletion in gastric carcinoma. *Genes Chromosomes Cancer*. **50**(6), 421–433 (2011). 21412930 10.1002/gcc.20867

[26] Engstrom, L. D. et al. MRTX1719 is an MTA-cooperative PRMT5 inhibitor that exhibits synthetic lethality in preclinical models and patients with MTAP-deleted cancer. *Cancer Discov*. **13**(11), 2412–2431 (2023). 37552839 10.1158/2159-8290.CD-23-0669 PMC10618744

[27] Fedoriw, A. et al. Anti-tumor activity of the type I PRMT inhibitor, GSK3368715, synergizes with PRMT5 inhibition through MTAP loss. *Cancer Cell.* **36**(1), 100–114 (2019). 31257072 10.1016/j.ccell.2019.05.014

[28] Cottrell, K. M. et al. Discovery of TNG908: A selective, brain penetrant, MTA-cooperative PRMT5 inhibitor that is synthetically lethal with MTAP-deleted cancers. *J. Med. Chem.* **67**(8), 6064–6080 (2024). 38595098 10.1021/acs.jmedchem.4c00133 PMC11056935

[29] Rodon, J. et al. First-in-human study of AMG 193, an MTA-cooperative PRMT5 inhibitor, in patients with MTAP-deleted solid tumors: Results from phase I dose exploration. *Ann. Oncol.* **35**(12), 1138–1147 (2024). 39293516 10.1016/j.annonc.2024.08.2339

[30] Vieito, M. et al. Phase 1 study of JNJ-64619178, a protein arginine methyltransferase 5 inhibitor, in advanced solid tumors. *Clin. Cancer Res.* **29**(18), 3592–3602 (2023). 37491846 10.1158/1078-0432.CCR-23-0092

[31] Rodon, J. et al. A phase I study to evaluate the safety, pharmacokinetics, and pharmacodynamics of PF-06939999 (PRMT5 inhibitor) in patients with selected advanced or metastatic tumors with high incidence of splicing factor gene mutations. *ESMO Open.* **9**(4), 102961 (2024). 38640748 10.1016/j.esmoop.2024.102961 PMC11047177

[32] Barekatain, Y. et al. Homozygous MTAP deletion in primary human glioblastoma is not associated with elevation of methylthioadenosine. *Nat. Commun.* **12**(1), 4228 (2021). 34244484 10.1038/s41467-021-24240-3 PMC8270912

[33] Brune, M. M. et al. MTAP as an emerging biomarker in thoracic malignancies. *Lung Cancer*. **197**, 107963 (2024). 39357262 10.1016/j.lungcan.2024.107963

